# Excretory/Secretory Metabolome of the Zoonotic Roundworm Parasite *Toxocara canis*

**DOI:** 10.3390/biom10081157

**Published:** 2020-08-06

**Authors:** Phurpa Wangchuk, Owen Lavers, David S. Wishart, Alex Loukas

**Affiliations:** 1 Centre for Molecular Therapeutics, Australian Institute of Tropical Health and Medicine, James Cook University, Cairns, QLD 4878, Australia; alex.loukas@jcu.edu.au; 2 Earville Vets, 474 Mulgrave Road, Cairns, QLD 4870, Australia; owenlavers@gmail.com; 3 Department of Biological Science, University of Alberta, Edmonton, AB T6G 2E9, Canada; dwishart@ualberta.ca

**Keywords:** helminths, gastrointestinal nematode, *Toxocara canis*, toxocariasis, excretory-secretory products, small molecules, metabolomics

## Abstract

Toxocariasis is a zoonotic disease affecting humans that is predominantly caused by *Toxocara canis* and *T. cati*, primarily parasites of dogs and cats, respectively. *Toxocara* generally establishes long-term infections by co-opting its host’s physiological processes, while at the same time exploiting the nutritional environment. Adult stage *T. canis* reside in the gut of the definitive canine host where they employ a suite of strategies to combat intestinal immune responses by actively producing and releasing excretory-secretory products (ESPs). The protein component of *T. canis* ESPs has been widely studied, but characterisation of the non-protein ESP complement remains neglected. To characterize the secreted metabolome of *Toxocara* ESPs and to shed light on the parasite’s metabolic processes, we profiled the ESPs of *T. canis* using both gas chromatography (GC) and liquid chromatography (LC) mass spectrometry approaches. We successfully identified 61 small molecules, including 41 polar metabolites, 14 medium-long chain fatty acids (MLCFAs) and six short chain fatty acids (SCFAs). We identified talose, stearic acid and isovalerate as the major compounds belonging to the polar, MLCFA and SCFA chemical classes, respectively. Most of the 61 identified metabolites appear to have been produced by *T. canis* via three distinct metabolic pathways - fatty acid, amino acid and carbohydrate metabolism. The majority of the identified ESPs have known biological properties, especially as immunomodulators. However, there is limited/no information on the biological roles or applications of 31 ESP biomolecules, suggesting that these may have novel activities that merit further investigation.

## 1. Introduction

Soil-transmitted helminths (STHs) infect and adversely affect billions of people and animals worldwide [1]. One of the most widespread public health and economically important zoonotic STH infections is toxocariasis. The clinical syndromes associated with human toxocariasis include visceral larva migrans, ocular larva migrans and covert toxocariasis [2]. Covert toxocariasis is most frequently found in children and the clinical symptoms include fever, headaches, abdominal pain, cervical lymphadenitis and hepatomegaly. On the other hand, patients with visceral larva migrans are mostly asymptomatic, while patients with ocular larva migrans may have chronic inflammation and permanent eye damage [3]. *Toxocara canis* and *T. cati* are parasites of dogs and cats, respectively, but can accidentally infect humans and are the most common causes of human toxocariasis [4]. Although human toxocariasis remains primarily a soil-transmitted zoonosis, cases of infection occurring after playing with pet animals, consumption of contaminated raw vegetables, geophagia (eating soil or sand) or consumption of undercooked meat or offal from potential paratenic homeotherm hosts have been identified as possible risk factors for acquiring toxocariasis [5,6,7,8,9]. For these reasons, toxocariasis has been reported in both developed and developing countries, with tropical regions being the hotspots of this infection [2]. For example, human seroprevalance of toxocariasis in European countries (France, the Czech Republic and Austria) ranges from 2 to 44%, with higher values in rural areas, whereas more than 63% (Indonesia) to 93% (La Reunion) seroprevalence has been reported in less developed tropical countries [10]. The wide distribution and seroprevalence estimates for human toxocariasis worldwide is beginning to raise considerable public health concerns. It calls for increased efforts to understand *Toxocara* parasites and improved measures to prevent the adverse health effects of toxocariasis [11].

Over the past decade, significant progress has been made in understanding the genomics, transcriptomics, proteomics and molecular biology of *T. canis* [11,12,13,14,15]. During infection, the ingested *Toxocara* eggs containing infective larvae of *T. canis* penetrate the small intestinal wall of the host and migrate through the circulatory system to various organs including the liver, lungs, central nervous system, and other tissues [11]. The infective larval stage has an extraordinary ability to survive for many years in the tissues of diverse vertebrate species, as well as to develop to maturity within the intestinal tract of its preferred canid host. The survival of *T. canis* in the host can be attributed to the synthesis and release of excretory-secretory products (ESPs) that are required by: (i) infective larval stage for tissue penetration, migration, feeding and modulation of host immunity; and (ii) adult stage worms for establishing chronic infection in the definitive host gastrointestinal tract. *T. canis* employs multiple molecular and physical strategies to manipulate and evade the immune system, including the formation and constant turnover of a specialised mucin-rich surface coat on the larva’s epicuticle that can shed when under immune attack and enable the parasite to evade host immune responses [16]. The antigenic ESPs released by *T. canis* infective stage under in vitro culture conditions are used for serodiagnosis (enzyme-linked immunosorbent assay) of human toxocariasis [11]. Proteomic analysis of the antigenic ESPs of *T. canis* infective larval stage has resulted in the identification of 19 proteins [12], of which a family of secreted mucin-like glycoproteins bearing multiple glycan side-chains is predominant [17]. Adult *T. canis* ESPs, on the other hand, consist of 870 detectable proteins [13].

While the proteinaceous ESP components are well characterised from the ESPs of the infective larval and adult stages of *T. canis*, there is a significant knowledge gap with regard to the non-proteinaceous small biomolecules in the ESP complement. To devise effective control and treatment strategies for *Toxocara* infection, it is important to comprehensively study the entire molecular ESP content to better understand the profile and characteristics of the compounds, and how *T. canis* infects and manipulates its host. Indeed, it is already known that the O-glycans are produced by the infective stages of *Toxocara* species [18,19]. In a closely related human roundworm, *Ascaris lumbricoides*, compounds such as ecdysones facilitate moulting, while dimethylheptatriacontanes are involved in sexual communication [20]. Similarly, it is likely that *T. canis* also produces small biomolecules responsible for specific biological functions and may have distinct roles in signalling, inflammation, tissue invasion and immune suppression. Identifying the small biomolecules from the infective larval and adult stages of *T. canis* ESPs could lead to improved insights into its metabolism, survival and competition mechanisms [21]. These advances could accelerate the development of effective diagnostic tools and anti-parasitic drugs to combat *Toxocara* infections. Indeed most drugs used to treat infectious diseases exploit fundamental differences between the host’s and pathogen’s metabolism by differentially targeting those enzymes in the pathogen that are absent or highly divergent from those found in the host [22,23]. Understanding key *Toxocara* ESP compounds will be crucial to developing new and effective anti-parasitic drugs. In this regard, metabolomics techniques offer a powerful route towards better understanding the metabolism of *T. canis* and discovering potential marker compounds that could be used to develop effective diagnostics, therapeutics, and even vaccines [14,15].

Due to the difficulty and ethical sensitivities around working with dogs, we had limited access to parasite material. We therefore collected only live adult stage parasites (both sexes combined) from euthanised canine hosts (impound dogs collected for laboratory demonstration for veterinary students) that were naturally infected with *T. canis*, cultured them in a single component culture medium, and analysed their ESPs via gas chromatography-mass spectrometry (GC-MS) and liquid chromatography–mass spectrometry (LC-MS). We then used extensive electronic database and literature searches to identify the potential immunomodulatory roles that these compounds may play in the biology or infectivity of *Toxocara* parasites.

## 2. Materials and Methods

### 2.1. Collection of Toxocara canis ESPs and Sample Preparation

Adult *T. canis* parasites were collected from euthanised stray/impounded dogs in Cairns, Australia. The small intestine was dissected longitudinally and the worms were carefully collected using tweezers and then washed three times with phosphate buffered saline (PBS) containing 5% antibiotic/antimycotic (AA) to remove faecal debris and other microbial components. The worms (sexes unidentified) were then transferred to six petri dishes (two worms/petri dish—each dish containing 25 mL of culture media with 2%AA), which represented 6 biological replicates named TC-1, TC-2, TC-3, TC-4, TC-5, TC-6. The parasites were cultured in a CO_2_ incubator (SANYO, Moriguchi, Japan) at 37 °C in 5% CO2 for 2 h to allow regurgitation/defecation of host-derived products that constitute the worm’s nutritional source. The initial ESP material was discarded, and the live worms were fed with fresh culture media. ESP in each biological replicate was removed/collected and centrifuged at 1831× *g* for 20 min at 4 °C to remove eggs and debris. The supernatant was then filtered using a 10 kDa cut-off Amicon ultra centrifugal filter (Merck Millipore, Sydney, Australia, 15 mL) to remove macromolecules such as proteins, large carbohydrates or polynucleotides. The filtrates (<10 kDa) formed the six biological replicates for small molecule ESPs of *T. canis*.

### 2.2. Cryomill Extraction of ESP Samples for GC-MS Analysis

We used previously described methods [1,24] for ESP sample preparation for follow-on GC-MS analysis. Briefly, the six ESP biological replicates (20 mg) from *T. canis* were placed in pre-chilled cryomill tubes containing a methanol:water (3:1, *v*/*v*, 600 µL) solution with ^13^C-sorbitol and ^13^C,^15^N-valine (Sigma-Aldrich, Sydney, Australia) as isotopically labeled internal standards. Each sample was extracted using a Precellys 24 Cryolys unit (Bertin Technologies, Rue Louis Armand ZI, France) at −10 °C and 5179× *g* (3 × 30 s pulses, 45 s intervals between pulses). Pre-chilled chloroform (150 µL) was then added to the homogenate (600 µL) in a fresh microfuge tube and the solution was vortexed vigorously to obtain a monophasic mixture of chloroform:methanol:water (1:3:1, *v*/*v*). The mixture was chilled on ice for 10 min with regular mixing, then centrifuged (1500× *g*) for 5 min (0 °C), and the supernatant was transferred to a fresh microfuge tube (1.5 mL). Milli-Q water (300 µL) was added to each sample tube to obtain a biphasic partition of the solution (chloroform:methanol:water 1:3:3, *v*/*v*), then each tube was vortexed vigorously, and then centrifuged again for a further 5 min at 0 °C. Both the upper aqueous phase (methanol:water) and the lower phase (chloroform) were collected separately. Any remaining solution (aqueous or chloroform) was pooled to obtain a pooled biological quality control (PBQC), which was used to monitor the variation arising from the instrumental analysis. A total of 2 × 18 = 36 samples were prepared and labelled including 12 (6 aqueous and 6 chloroform) as ‘*T. canis* ESP’ (TC-1 to 6), 12 as ‘PBQC quality control’ (PBQC-1 to 6), and 12 as ‘media control’ (MED-1 to 6). All of these samples were then derivatised for further GC-MS analyses (see below).

### 2.3. Targeted Analysis of Polar Metabolites Using GC-MS

Sample derivatisation was performed as reported earlier for the GC-MS metabolomic analysis of ESPs derived from hookworms, whipworms and tapeworms [1,24,25]. The upper aqueous phase composed of 3:1 methanol:water (~900 µL) was aliquoted (50 µL) into a pulled point insert, dried using an evaporator at 30 °C (Christ RVC 2-33 CD, John Morris Scientific Australia) and then anhydrous methanol was added (50 µL). The solutions were then derivatised by adding methoxyamine (20 µL, 30 mg/mL in pyridine, Sigma Aldrich/Merck) and *N*,*O*-Bis(trimethylsilyl) trifluoroacetamide (BSTFA) + 1% trimethylchlorosilane (TMCS) (20 µL, ThermoFisher Scientific Deepdene, Victoria, Australia) at 37 °C for 30 min. Each sample was allowed to sit for 2 h to complete the derivatisation reaction. The derivatised polar sample (1 µL) was injected into an Agilent (Victoria, Australia) 7890 GC-MS (5973 MSD), which used an Agilent VF-5 ms column (30 m × 0.25 mm × 0.25 µm) [26]. The oven temperature was set to 35 °C and then slowly ramped to 325 °C (at 25 °C/min) and held for 5 min. Helium (carrier gas) was set to flow at a rate of 1 mL/min. GC-MS chromatograms were collected across the mass (*m*/*z*) range of 50–600 atomic mass units (amu).

Agilent’s Mass Hunter Quantitative Analysis Software (v.7) and the Metabolomics Standard Initiative Level 1 confirmation were used for identifying the compounds. Each chromatogram peak represented a compound and this compound was identified by matching an observed Electron Impact Ionization-Mass Spectrometry (EI-MS) spectral ion peak with EI-MS spectra in the Mass Hunter Library (Agilent). The EI-MS spectral ion peak library in the Mass Hunter Library was developed by running standards prior to this experiment. The relative concentration of each metabolite was determined by comparing the metabolite peak areas with the peak areas of the isotopically labelled internal standards (13C-sorbitol and 13C 15N-valine). The compound data matrix of the six ‘*T. canis* ESP’ biological replicates were converted to a format (.csv) compatible as the input file for MetaboAnalyst 4.0 (https://www.metaboanalyst.ca/) to perform further univariate and multivariate data analysis.

### 2.4. Targeted Analysis of Medium–Long Chain Fatty Acids (MLCFAs) Using GC-MS

The chloroform/organic layer of the ESPs and control samples was analysed via a GC-MS protocol described by us earlier [24,25]. In this protocol, the organic/chloroform phase of each biological replicate sample was sealed into GC vials and trans-esterified to generate fatty acid methyl esters. Samples were then mixed with 5 μL Methprep-II (Alltech, Deerfield, IL, USA ) using a Gerstel MPS2 autosampler robot whereupon the solutions were incubated for 30 min at 37 °C with slow shaking. Small aliquots of these samples (2 μL) were injected into an Agilent 7890 gas chromatograph (−70 eV, split mode 10:1, helium flow at 1.5 mL/min, inlet set at 250 °C), which was connected to a SGE BPX70 column (60 m × 0.25 mm i.d. × 0.25 um film thickness, Trajan Scientific and Medical, Ringwood, Australia) and a triple quadrupole mass selective detector (Agilent Technologies, Mulgrave, Victoria, Australia). The oven temperature was initially set to 70 °C for 1 min, then gradually increased to 150 °C (40 °C/min), 200 °C (4 °C/min), 220 °C (3 °C/min), and finally held at 250 °C for 4 min. ^13^C18:0 was used as an internal standard. The mass spectrometer scan mode was set to *m*/*z* 50–450 (3.6 scans/s, M^+^ mode). Target ion peaks/areas were extracted using an in-house MHFAL (Mass Hunter Fatty Acid Library, Agilent) and the compounds were identified by a library matching technique. The output data matrix was converted to compatible format (.csv) for further analysis via MetaboAnalyst 4.0.

### 2.5. Targeted Analysis of Short Chain Fatty Acids Using LC-MS

Using previously described LC-MS protocols [24,25], we derivatised a small portion (20 µL) of the organic phase of the ESP extract (prepared using the ‘Cryomill extraction method’) using a 3-nitrophenylhydrazine (3-NPH), which converted the short chain fatty acids (SCFAs) to their 3-nitrophenylhydrazones. The samples (1 µL in triplicate) were then injected into a Shimadzu LC 30AD-TQ 8060 triple quadrupole mass spectrometer (SHIMADZU Scientific Instruments Inc., Columbia, MD, USA) connected to an Agilent EC-C18 poroshell 120 column (50 mm × 2.1 mm × 2.7 µm). A two-component (A:B) gradient LC separation was performed with the mobile phase A being 100% water (+0.1% formic acid) and mobile phase B being 100% acetonitrile (+0.1% formic acid). The column flow rate and temperature were set to 0.55 mL/min and 40 °C, respectively. Each sample total run time was 17 min with an elution gradient of 15% B for 1 min, 15–25% B for 7 min, 25–30% for 1 min, 30–100% for 3 min, held at 100% for 3 min, then back to starting conditions and held for 2 min. Authentic standards for acetate, propionate, isobutyrate, butyrate, 2-methylbutyrate, isovalerate, valerate, 3-methylvalerate, isocaproate, and caproate were used as reference standards in concert with multiple reaction monitoring (MRM) to assist with compound identification. Collision energies for each analyte were optimised (22 eV, 25 eV, and 28 eV) and selected according to their sensitivity and the compounds were identified (as described above) using the positive mode [M^+^] ion. The output data matrix was converted to csv format for further analysis via MetaboAnalyst 4.0.

### 2.6. Metabolite Identification Standards and Data Analysis

Small biomolecules were identified and confirmed according to the metabolomics standard initiative (MSI) definitions of level 1 & level 2 identification [24]. The Metabolomics Australia metabolite spectral library was developed using authentic standards for both GC-MS and LC-MS prior to running the *Toxocara* samples. For statistical analysis and data interpretation, we used the freely available online software package MetaboAnalyst [27]. The input data matrix (in csv format) contained information regarding the identified metabolites and their peak areas. This input was used by MetaboAnalyst 4.0 to perform various univariate and multivariate statistical analyses. MetaboAnalyst, by default, replaces all the missing and zero values (which are typically caused by low abundance metabolites) with a small value that is assumed to be below the detection limit. This helps with data normalisation and prevents divide-by-zero errors. The data was median normalised prior to performing univariate analysis, volcano plot analysis, multivariate principal component analysis (PCA) and heatmap analysis.

### 2.7. Literature Assessment of the Small Molecules Identified from Toxocara canis ESPs

The list of small molecules identified via GC-MS and LC-MS from the ESPs of *T. canis* was generated and a comprehensive literature survey was conducted for each compound. We used ‘The human metabolome database’ (HMDB), which contains 114,100 metabolite entries [28], ‘DrugBank’, which contains information on 2280 drugs and drug metabolites [29], and ‘PubChem’, which contains data on 94,017,529 compounds [30]. Information on the chemical structures, compound identifier numbers, chemical properties, biological activities, patents, health, safety, and the toxicity data were crosschecked and recorded. In addition to these databases, Google Scholar and SciFinder Scholar were used for tracing additional references on the biological activities and pharmaceutical roles of each compound. The information was then tabulated.

## 3. Results

We collected live adult *T. canis* from freshly euthanised dogs, cultured them in a single component Glutamax culture media, prepared the small molecule ESP extract (<10 kDa centrifugal filter), lyophilised them, fractionated them into polar and non-polar fractions, derivatised them and ultimately analysed them using GC-MS and LC-MS. Using GC-MS, we identified 41 polar metabolites and 14 medium-long chain fatty acids. Using LC-MS, we identified six SCFAs. Each metabolite was then annotated through careful searches in the literature and databases to identify their metabolic pathways and reported biological or non-biological functions/roles/properties.

### 3.1. Polar Metabolites of Toxocara canis ESPs Identified Using GC-MS

From the polar fraction of the ESP extracts, we identified 41 polar metabolites using a targeted GC-MS analysis. Identified compounds ranged in mass (*m/z*) from 89 to 342 amu. Of the 41 metabolites detected, talose (mass of 180) was identified as the most abundant component of the ESPs (see Table 1), which was followed in relative abundance by (based on their peak areas) glucose, sorbitol, mannitol, lactic acid, succinic acid, fructose, maltose, xylose and gluconic acid. Scyllo-inositol and methionine were present only sparingly in the polar fraction analysed here.

### 3.2. Reported Biological Activities of Polar Metabolites

Based on our literature review, we found that of the 41 metabolites identified from *T. canis* ESPs (listed in Table 1), biological activities of 20 polar metabolites were reported previously, but there was a lack of information on the other 21 polar metabolites. Of the 20 polar metabolites known to have various biological properties, eight of them have been indicated to possess anti-inflammatory activities. Many metabolites have been indicated to have multiple biological properties, as described in Table 1. For example, GABA is reported to control blood pressure, reduce blood sugar in diabetic patients [39], and is a wound healing agent [40]. Another major metabolite, talose, is a rare sugar and has been less explored for its biological activities. However, talose was reported to inhibit the growth of *Caenorhabditis elegans* [31].

### 3.3. Univariate Volcano Plot Analysis of Polar Metabolites

**Figure 1:**
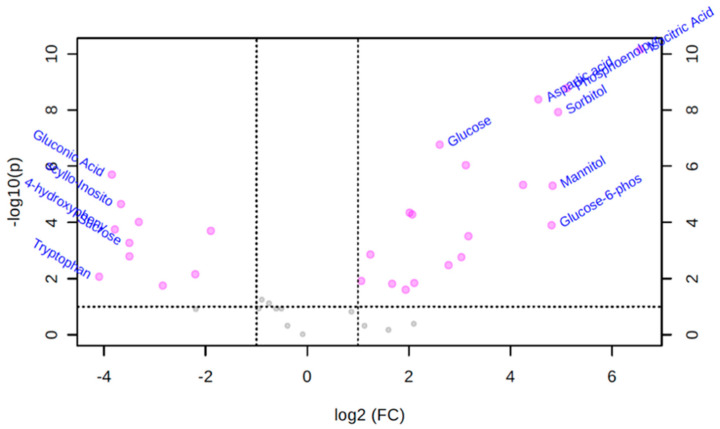
Important features selected by volcano plot analysis with a fold change threshold (*x*) of 2 and a *t*-test threshold (*y*) of 0.1. The red circles represent features above the threshold. Both fold changes and log transformed fold change for each significant compound are shown in the extended table of the volcano plot.

A volcano plot is a univariate analysis technique that provides a preliminary overview about features that are potentially significant (indicated by fold change above threshold for each variable) in discriminating the conditions under study. A variable is reported as significant if this number of pairs is above a given count threshold (default >75% of pairs/variable). The purpose of the fold change measurement is to compare absolute value changes between two group means. The fold change is normally plotted using a log2 scale so that same fold change (up or down regulated) will have the same distance to the zero baseline. Figure 1 shows the important compounds with log transformed fold changes identified by the volcano plot when *T. canis* polar metabolite features were compared to culture media features. The volcano plot showed that 29 metabolites from the *T. canis* ESPs were significantly different from culture media-derived metabolites (fold change >2, *p*-value < 0.05) in their peak intensities (Figure 1). The five polar metabolites that showed the most significant positive fold-changes were isocitric acid (log2 FC 6.55), phosphoenolpyruvate (log2 FC 5.11), sorbitol (log2 FC 4.94), mannitol (log2 FC 4.83), and glucose-6-phosphate (log2 FC 4.81).

### 3.4. Multivariate Chemometric Analyses of Polar Metabolites

**Figure 2:**
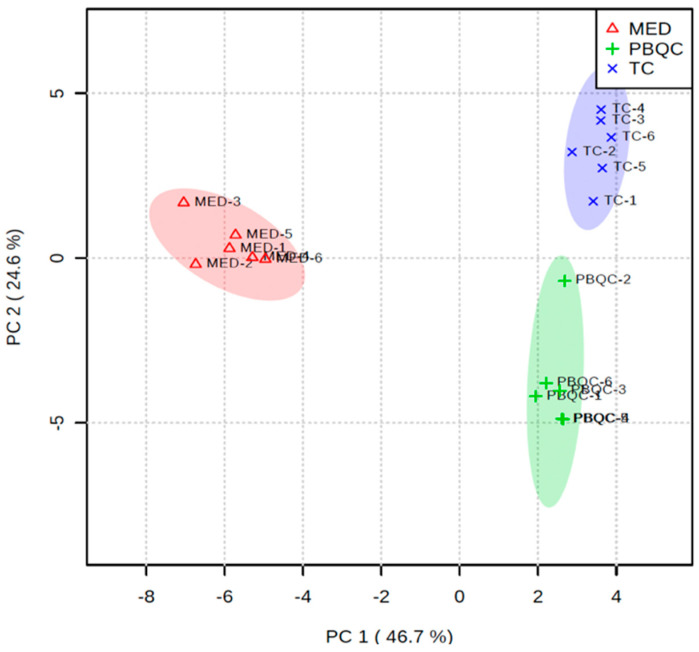
Principal component analysis of polar metabolites of *Toxocara canis* excretory-secretory products (ESPs) showing clear separation from the media component. MED—culture media samples, TC—*T. canis* ESP samples, PBQC—pooled biological replicates for quality control (pooled from the biological replicates of *T. canis* ESPs and culture media samples). Data analysed using MetaboAnalyst 4.0 (*N* = 6 replicates).

In addition to the univariate volcano plot analysis described above, we performed multivariate statistical analysis to compare the polar metabolites (as measured by GC-MS) present in *T. canis* ESPs and culture media (Glutamax). The principal component analysis (PCA) of the three sample groups (*T. canis* ESPs, culture media and PBQC) showed clustering of biological replicates within their sample groups. There was clear separation between the groups of biological replicates of *T. canis* ESPs and the culture media, indicating that the *T. canis* metabolites are distinct from the metabolites detected in the culture media (Figure 2). The PBQC is a quality control group pooled from the biological replicates of *T. canis* ESPs and culture media samples. Since PBQC also showed good clustering and its alignment fell in between the *T. canis* ESPs and culture media groups, this indicated that the quality of the replicates was good.

**Figure 3:**
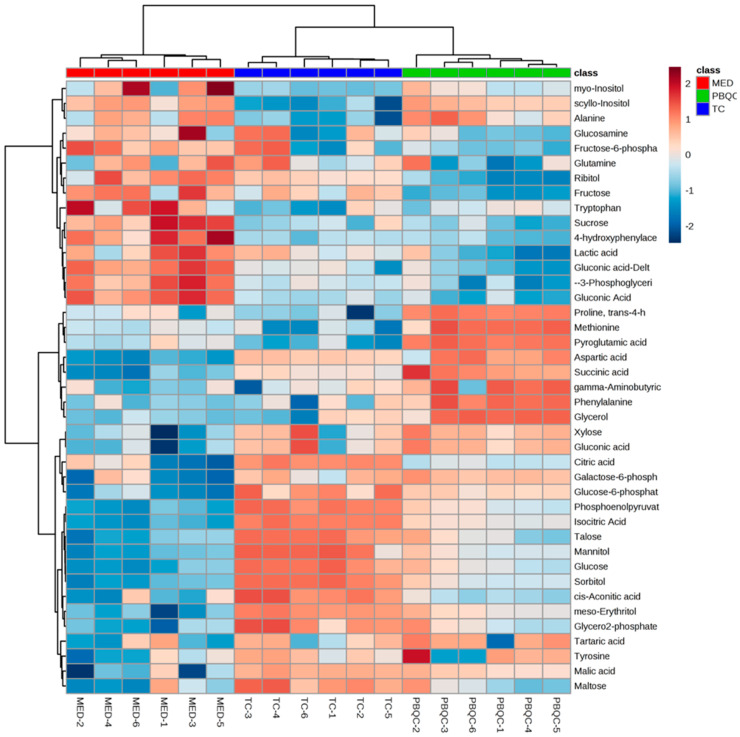
Heat map of polar metabolites of *T. canis* ESPs showing metabolite patterns by groups. The deep brown color indicates the highest concentrations (based on peak intensities/areas) and the deep blue indicates lowest or negligible concentrations. MED—culture media samples, TC—*T. canis* ESP samples, PBQC—pooled biological replicates for quality control (pooled from the biological replicates of *T. canis* ESPs and culture media samples). Data analysed using MetaboAnalyst 4.0 (*N* = 6 replicates).

A heat map reflecting the abundance of the different metabolites for the three different sample groups was generated (Figure 3). The heat map showed clear differences in the compositions of the different sample groups. For example, talose, isocitric acid, glucose, mannitol and sorbitol were the most abundant polar metabolites in the T. *canis* ESPs. On the other hand, the culture media was particularly rich in gluconic acid, lactic acid, 3-phosphoglyceric acid, 4-hydroxyphenylacetic acid and sucrose.

### 3.5. Non-Polar Components of T. canis ESPs

The non-polar components isolated via chloroform extraction from the ESPs included both medium-long chain fatty acids (MLCFAs) as well as short-chain fatty acids. These are described in more detail below.

#### 3.5.1. Medium–Long Chain Fatty Acids Identified Using GC-MS

From the non-polar extract of *T. canis* ESPs, we identified 14 MLCFAs (C10–C22), which included saturated and un-saturated fatty acids with masses ranging from 173–328 Da (see Table 2). Stearic acid (C18:0) was putatively identified as the major fatty acid within the non-polar extract of the ESPs (based on peak intensity), which was followed by palmitic acid (C16:0) and myristic acid (C14:0). Margaric acid (C17:0) is the least abundant saturated fatty acid in the *T. canis* ESPs. Four unsaturated fatty acids were also identified in the ESPs, including oleic acid (C18:1 9Z-cis), linoleic acid (C18:2), arachidonic acid (C20:4) and docosahexaenoic acid (C22:6).

#### 3.5.2. Short Chain Fatty Acids Identified Using LC-MS

Using LC-MS, we identified six SCFAs in our ESPs including isovalerate (3-MBA, C5:0, peak area = 603,922), 2-methylbutyrate (C4:0, peak area = 354,798), isobutyrate (C4:0, peak area = 217,617), acetate (C2:0, peak area = 185,327), propionate (C3:0, peak area = 29,039) and butyrate (C4:0, peak area = 27,033). Isovalerate was the most abundant SCFA while butyrate was the least abundant SCFA.

### 3.6. Potential Biological Properties of Non-Polar Metabolites

In similar fashion to polar metabolites, we conducted a literature review on each non-polar metabolite listed in Table 2 for their biological properties. We found that seven non-polar MLCFAs including lauric acid (C12:0), palmitic acid (C16:0), stearic acid (C18:0), oleic acid (C18:1, 9Z- *cis*), linoleic acid (C18:2), arachidonic acid (C20:4) and docosahexaenoic acid (C22:6) had been previously studied for their biological activities. Six MLCFAs (Table 2) and three SCFAs including acetate, propionate and butyrate were reported to possess anti-inflammatory properties [25]. While the most abundant saturated fatty acid, stearic acid, is known to possess anti-inflammatory activities; isovalerate, the major SCFA found in *T. canis* ESP had limited information on its biological properties.

## 4. Discussion

*Toxocara canis* is one of the most ubiquitous zoonotic parasites and is particularly prevalent in tropical countries. In humans it causes toxocariasis, including visceral larva migrans, ocular larva migrans and covert toxocariasis [2]. Diagnosis of human toxocariasis has been challenging due to its relatively nonspecific and mild symptoms, which include diarrhoea, vomiting, nasal discharge, weight loss, nutritional deficit and stunting, and bulging of the abdomen [54]. Diagnostic techniques include microscopic or macroscopic examination of the parasite, various DNA analysis techniques, and a variety of immune-based serological assays. Serological assays including enzyme-linked immunosorbent assay (ELISA), dot-ELISA, recTES-30 ELISA, and immunoblot are considered gold standard techniques for laboratory diagnosis of human *Toxocara* infection [3]. However, their sensitivities and cross-reactivities differ from place to place and they are often affected by the presence of infections by other parasites. This suggests that other diagnostic techniques should be considered, especially those that use more reliably measured chemical markers. Studies on other parasitic nematode ESPs have revealed the presence of diagnostically unique nucleic acids, glycans, lipids, and non-proteinaceous small biomolecules [25,55,56]. Therefore, characterising the metabolome of *T. canis* ESPs and identifying specific metabolites would be valuable for developing more sensitive diagnostics and biomarkers of infection, as well as potential therapeutics that exploit the immunoregulatory properties of helminth ESPs [57,58].

Metabolite studies with parasitic helminths, however, face several technical challenges. First, it is often difficult to obtain sufficient quantities of worms from the natural host, which limits ESP collection. Second, the metabolomes of parasites can be influenced by strains and circadian rhythm of the host, sex of the parasite, timing of isolation from the host, culture media and metabolomic techniques and tools used for the study. Third, the removal of a parasite from its host for in vitro experiments impacts the state of dynamic equilibrium with their hosts, and the metabolite composition might differ between in vivo and in vitro culture settings. Fourth, the physiology of worms and the identified metabolites secreted by the parasite in vivo, or even in co-culture with host cells or serum, makes it difficult to determine the origin of identified compounds as host-, parasite- or even media-derived. In this study, we collected adult *T. canis* from the natural host (impound dogs) and analysed their ESPs for metabolites. We demonstrated that *T. canis* produces a large number of non-proteinaceous small biomolecules. Using targeted GC-MS and LC-MS, we identified 61 small biomolecules in total: 41 polar metabolites, 14 MLCFAs, and 6 SCFAs. The majority of these metabolites seem to arise through fatty acid, amino acid and carbohydrate metabolism that the parasite engages in during the ESP culturing process. While talose was found to be the major polar metabolite, stearic acid and isolvalerate were identified as the major MLCFA and SCFA present in the ESP of *T.canis*. Talose is a relatively rare and unnatural monosaccharide and is a C-2 epimer of galactose. This fact potentially makes talose a very useful biomarker of *T.* *canis* infection. However, it is not clear whether *T. canis* produces talose as a natural metabolite or whether the talose production is an artefact or by-product of the media the parasite is cultured in. Further studies will be needed to confirm the presence of talose in infected animals or infected tissues/biofluids. If this can be confirmed using live host infection, we would argue that a ‘talose test’ would be a fast and simple way of detecting *T. canis* infection. Six of the 41 polar metabolites (alanine, phenylalanine, methionine, aspartic acid, glutamine and glucose) were identified previously from somatic tissue extracts of *T. canis* [59] using NMR. In particular, previous NMR studies identified 17 amino acids, two organic acids, two sugars, and two phosphorus metabolites from the somatic tissue extract. However no fatty acids or SCFAs were reported in this earlier study [59]. It has been suggested that helminths can produce SCFAs de novo, and d-glucose-^14^C labelling studies using *Ancylostoma caninum* (a hookworm nematode) have shown that glucose is metabolised to produce acetate, propionate, and CO_2_ [25]. In addition, a recent review [60] suggested that the formation and excretion of acetate as a metabolic end product of energy metabolism occurs in numerous protist and helminth parasites. Likewise, this study showed that SCFAs may have been produced by *T. canis* as an end product of energy metabolism (using glucose). Since we used PBS with 5% antibiotic/antimycotic to remove faecal debris and other microbial components and culture media with 2% antibiotic/antimycotic for worm culture, we expected minimal microbial contamination. On the other hand, the contribution of the commensal microbiota in the mammalian gut to the production of SCFAs is well known [61]. Given that the production of SCFAs by helminths remains refutable, it is unlikely that the presence of SCFAs can be used to diagnose *T. canis* infections conclusively, nor would SCFAs be likely to serve as metabolic targets to limit their infection.

Interestingly, we detected many of the same *T. canis* metabolites, including SCFAs, in the ESPs of other nematodes including *A. caninum* [25] and the tapeworm *Dipylidium caninum* [1], which were isolated from the same canine hosts. For instance, *A. caninum* ESPs contained pyroglutamic acid and propionate (as well as other SCFAs) as the major constituents of their polar ESP metabolome [25]. *D. caninum* ESPs contained succinic acid as the major polar metabolite, while acetate was the only identified SCFA [1]. On the other hand, *T. canis* ESPs contain talose and isovalerate as the major constituents of the polar and SCFA fractions, respectively. Ten polar metabolites (citric acid, glutamine, cis-aconitic acid, glucose-6-phosphate, glucosamine, galactose-6-phosphate, fructose-6-phosphate, gluconic acid, sucrose and tryptophan) appear to be exclusively produced by *T. canis*, as these were absent in *A. caninum* ESPs generated in the same basal media (Glutamax) using the same culturing processes. *D. caninum* ESPs also did not contain glucosamine, fructose-6-phosphate, gluconic acid, gluconic acid-δ-lactone, 3-phosphoglyceric acid and tryptophan, all of which were present in the ESPs of *T. canis*. These variances in ESP metabolite composition were expected since they belong to different phyla of parasitic helminths and they compete for the same nutritional sources in the gut. They also potentially defend themselves (or their privileged intestinal niche) against each other within the same canine host. Indeed, it is possible that helminths use their ESPs as “bioweapons” to guard their habitat against their competitors. The intra- and interspecific competition among helminth parasites that share the same habitat for reproduction, growth and life cycle completion has been well documented in parasitic trematodes [62]. Similarly, our post-mortem observations within the canine hosts revealed that wherever *T. canis* was found in abundance, *A. caninum* was absent, and vice versa. This is likely reflective of their intraspecific competition. However, most of the parasite-specific metabolites that we identified herein may likely be playing a role only as a metabolic fuel, and there is nothing suggestive of any competitive advantage of *T. canis* over other helminths present in the same host. While it is tempting to hypothesise that mannitol may contribute to increased tolerance of parasites to salt, oxidative and osmotic stress as a result of mannitol’s function as a ‘compatible solute’, there is no evidence to date to support it.

A key limitation of this study is that our targeted approach of compound identification may have missed out many novel biomolecules (with no entries in the inhouse Mass Hunter library) that may play a more direct role in interspecies competition and growth suppression. In principle, some of these unidentified metabolites may be specifically used by *T. canis* to repel *A. caninum* and *D. caninum*. Further untargeted metabolomic studies of *T. canis* ESP are clearly warranted to identify these compounds. Ideally, these studies should be performed by analyzing the ESP samples (proteins and non-proteinaceous components) of different gastrointestinal helminths that polyparasitise the same canine host as opposed to studying single species in isolation.

Of the 61 small molecules identified in adult *T. canis* ESPs, there was limited/no information on the biological or physiological properties of 31 metabolites (including 21 polar compounds, 7 MLCFAs and 3 SCFAs). However, for at least 30 metabolites (including 20 polar compounds, 7 MLCFAs, 3 SCFAs) there was a good deal of previously known and well-supported data on their biological and physiological functions. We found that many of these better-annotated compounds were reported to possess anti-inflammatory, antimicrobial, antidiabetic, wound healing, laxative and anti-aggregant properties. They were also implicated in improving gut health barrier, as well as possessing anti-arhythmic, hypolipidemic, and vasodilatory properties, among others. Moreover, some of these compounds have been associated with prevention of coronary heart disease, hypertension, type 2 diabetes, rheumatoid arthritis, ulcerative colitis, Crohn’s disease and chronic pulmonary diseases [63] (see Table 1; Table 2). It is possible that these ESP compounds may be, individually or in combination, playing important roles in skewing the immune response of the infected host towards a phenotype that favours sustained immune suppression and “stealthy” growth of the *T. canis* parasite within the host. For example, the majority of the metabolites (17 metabolites) we identified were reported to possess anti-inflammatory properties. Therefore, it is highly likely that at least some of these molecules are used by *T. canis* for achieving its immune dampening effects on its host(s). In particular, the SCFAs, especially butyrate and propionate, are well-known anti-inflammatory agents. They have been implicated in the maintenance of colon homeostasis, in host defence against pathogenic microorganisms [61,64,65,66], and in the protection against autoimmune diseases [67,68]. It is possible that this combination (or cocktail) of anti-inflammatory metabolites found in *T. canis* ESPs may offer some direction/modality in the development of an all-natural, multi-metabolite therapeutic cocktail for treating a variety of gut disorders, such as inflammatory bowel disease (IBD), for which there is no cure.

While there are health benefits (such as reduced levels of IBD, irritable bowel syndrome (IBS) or other gut disorders) associated with helminth infections, extensive and chronic infection is a major cause or morbidity and even death in developing countries. Chronic *T. canis* infection is linked to stunted growth, decreased physical activity, and poor physical and mental development in children [69]. Moreover, high levels of methionine, which we detected in *T. canis* ESP, have been linked to infantile intellectual disability, delays in motor skills, sluggishness, muscle weakness, and liver problems [36]. It is possible that *T. canis* may be producing methionine in larger quantities while metabolising nutrients found in the host’s gut. Another metabolite produced by *T. canis* that appears to exhibit negative health affects is talose (a major metabolite of ESP). Talose is known to inhibit the growth of *Caenorhabditis elegans* [31], and given its widespread anti-metabolite role as an “unusuable” sugar analog, it may be involved in causing stunting in their host, which requires further corroboration. Since talose is a major component of the *T. canis* ESP, this compound could be exploited for developing specific diagnostic biomarkers to detect *T. canis* infections in humans and pets. The use of metabolite markers to detect bacterial or parasitic infections is not new. For instance, arachidonic acid can be used to detect and differentiate leprosy patients with low bacterial indices of *Mycobacterium leprae* from those with high bacterial indices [24]. Likewise, nitrite levels and heme levels in urine can be used clinically to detect the presence of bacteria causing urinary tract infections. Although metabolite biomarkers have yet to be applied for helminth diagnostics, their utility as specific diagnostic biomarkers will become more apparent as more helminth metabolomes are characterised. Further work is obviously needed to confirm the potentially utility of talose as a diagnostic marker for *T. canis* infection including assessments of whether this compound is detectable in host blood/urine or if it is up-/down-regulated in the host at the time of *T. canis* infection.

## 5. Conclusions

In this study, we identified at least 61 small molecules in *T. canis* ESP. These include 41 polar metabolites, 14 medium-long chain fatty acids, and six SCFAs. Most of these metabolites appear to be produced as a result of fatty acid, amino acid and carbohydrate metabolism that *T. canis* engages in during the ESP culturing process using a single component (glutamax) medium. It is possible that these metabolites, individually or in combination, are being used by the parasite to perform key biological functions in various facets of parasitism, including establishment of “stealthy” infection, immune suppression, intra-parasitic competition and protection from the host defences. Since the majority of the metabolites identified in *T. canis* ESP are known to possess anti-inflammatory properties, it is possible that this combination or “cocktail” of anti-inflammatory metabolites could be exploited to create an all-natural, fully biocompatible, anti-inflammatory cocktail to treat a plethora of inflammatory diseases (such as IBD or other immune dysregulation diseases). In addition to identifying a potentially useful therapeutic metabolite cocktail, we also identified a potentially diagnostic metabolite for *T. canis* infection. In particular, based on our preliminary work, we believe that talose (a major metabolite of *T. canis* ESP) could be exploited to develop a cheap, inexpensive *T. canis* specific diagnostic biomarker to detect *T. canis* infections in pets.

## Figures and Tables

**Table 1:** Polar metabolites identified from the ESPs of *T. canis* using GC-MS (the peak area of each *T. canis* ESP metabolite was subtracted from the peak area of media component).

Compound Name	Rt (min)	Mass (*m*/*z*, M^+^)	KEGG ID	Peak Area	Immunomodulatory Properties
Talose	10.82	180	C06467	6,433,882	Sakogochi et al. [31] showed that talose inhibited the growth of *Caenorhabditis elegans.*
Glucose	10.85	180	C00031	5,706,822	N/A
Sorbitol	10.99	182	C00794	4,350,846	Used as a laxative and irrigating solution (surgeries) [32].
Mannitol	10.95	182	C00392	3,751,129	Anti-edema and a diuretic agent [33].
Lactic acid	6.73	90	C00186	1,332,469	Immunosuppressant and anti-inflammatory [34].
Succinic acid	8.11	118	C00042	963,948	N/A
Fructose	10.73	180	C02336	120,152	Pro-inflammatory [35] and are associated with hypertension and liver disease [36].
Maltose	13.61	342	C00208	114,953	N/A
Xylose	9.86	150	C00181	90,585	N/A
Gluconic acid	11.26	196	C00257	90,577	N/A
Tyrosine	10.95	181	C00082	53,189	Prevents stress-induced depletion of norepinephrine and can cure biochemical depression [36].
Malic acid	9.01	134	C03668	46,865	Anti-aggregant and anti-inflammatory [37].
Glycerol-2-phosphate	10.11	172	C02979	46,569	N/A
meso-Erythritol	9.05	122	C00503	31,069	N/A
Citric acid	10.54	192	C00158	26,859	Antimicrobial, a natural preservative and cardioprotective [37].
Glucose-6-phosphate	12.33	260	n/a	22,966	N/A
Ribitol	10.09	152	C00474	22,726	N/A
Glucosamine	10.94	179	C00329	16,987	Anti-inflammatory [38] and used for the treatment of osteoarthritis [36].
Isocitric Acid	10.54	192	C00311	16,661	N/A
Gluconic acid-δ -lactone	10.84	178	C00198	16,341	N/A
Pyroglutamic acid	9.26	129	C01879	12,048	Sold as a “smart drug” for improving blood circulation in the brain [36].
myo-Inositol	11.65	180	C00137	11,665	Used as a treatment for polycystic ovary syndrome [39].
Sucrose	13.43	342	C00089	10,047	N/A
cis-Aconitic acid	10.25	174	C00417	6546	N/A
Galactose-6-phosphate	12.32	260	n/a	6029	N/A
Phosphoenolpyruvate	9.55	168	C00074	6003	N/A
Fructose-6-phosphate	12.38	260	n/a	4637	N/A
Aspartic acid	9.19	133	C00402	4042	Excitatory neurotransmitter [39].
(-)-3-Phosphoglyceric acid	10.48	186	C00197	3590	N/A
γ-Aminobutyric acid (GABA)	9.28	103	D00058	2096	Controls blood pressure, reduces blood sugar in diabetics [39], and it is a wound healing agent [40].
Tartaric acid	9.71	150	C02107	1927	N/A
Phenylalanine	9.42	165	C00079	1532	Anti-diabetic [41].
Alanine	6.93	89	C00041	1414	Anti-inflammatory [42,43,44] and alanine therapy has helped dissolve kidney stones in experimental animals [32].
Glycerol	10.12	92	C00116	1183	Anti-inflammatory [45].
Tryptophan	12.20	204	C00806	1179	Anti-inflammatory [46,47,48].
trans-4-Hydroxyproline	9.22	131	C05147	962	N/A
Glutamine	11.22	146	C00064	784	Anti-inflammatory [49]. Maintains gut barrier function and reduces septic morbidity and the symptoms of Irritable Bowel Syndrome [32].
4-Hydroxyphenylacetic acid	11.42	152	C00642	539	N/A
Gluconic acid	11.26	196	C00257	221	N/A
Methionine	9.22	149	C00073	81	Anti-inflammatory [50] and antioxidant [51]. Linked to infants’ intellectual disability, delays in motor skills, sluggishness, muscle weakness, and liver problems [36].
scyllo-Inositol	11.37	180	C06153	24	Transgenic mice fed with this compound was found to reverse memory deficits, reduce the amount of Aβ plaque in the brains of the mice and reversed other symptoms associated with it [36].

Rt = retention time. KEGG ID = information on the metabolism of a compound. ID = identity. N/A = not available.

**Table 2:** Medium–long chain fatty acids (MLCFAs) identified from *T. canis* ESP using GC-MS.

Fatty Acid Name	Rt (min)	Mass (*m/z*, M^+^)	KEGG ID	Peak Area	Biological Activities
Capric acid (C10:0)	7.68	172	C01571	9155	N/A
Undecylic acid C11:0	7.36	186	C17715	12,033	N/A
Lauric acid (C12:0)	9.21	200	C02679	11,585	Anti-inflammatory [24].
Tridecylic acid (C13:0)	9.01	214	N/A	13,614	N/A
Myristic acid (C14:0)	11.27	228	C06424	24,866	N/A
Pentadecylic acid (C15:0)	12.52	242	C16537	8505	N/A
Palmitic acid (C16:0)	13.83	256	C00249	358,443	Anti-inflammatory [52].
Margaric acid (C17:0)	15.26	270	N/A	6712	N/A
Stearic acid (C18:0)	15.25	284	C01530	565,394	Anti-inflammatory [53].
Oleic acid (C18:1, 9Z- *cis*)	17.15	282	C00712	1771	Anti-inflammatory and wound healing [24], insecticide and fungicide [36].
Linoleic acid (C18:2)	18.31	280	C01595	7507	Anti-inflammatory and wound healing [24].
Arachidic acid (C20:0)	19.61	312	C06425	11,388	N/A
Arachidonic acid (C20:4)	18.16	304	C00219	1035	Eicosanoids including prostaglandins, thromboxanes, and leukotrienes mediates inflammation [36]. It regulates platelet aggregation, blood clotting, smooth muscle contraction, leukocyte chemotaxis, inflammatory cytokine production and immune function [36].
Docosahexaenoic acid (C22:6)	26.83	328	C06429	3825	Anti-inflammatory [24]. It can reduce the level of blood triglycerides in humans, which may reduce the risk of heart disease [32].

Rt = retention time. KEGG ID = information on the metabolism of a compound. ID = identity. N/A = not available.
